# Evaluation of serological lateral flow assays for severe acute respiratory syndrome coronavirus-2

**DOI:** 10.1186/s12879-021-06257-7

**Published:** 2021-06-16

**Authors:** Bianca A. Trombetta, Savannah E. Kandigian, Robert R. Kitchen, Korneel Grauwet, Pia Kivisäkk Webb, Glenn A. Miller, Charles G. Jennings, Sejal Jain, Samara Miller, Yikai Kuo, Thadryan Sweeney, Tal Gilboa, Maia Norman, Daimon P. Simmons, Christopher E. Ramirez, Melissa Bedard, Catherine Fink, Jina Ko, Esmarline J. De León Peralta, Gerald Watts, Emma Gomez-Rivas, Vannessa Davis, Rocky M. Barilla, Jianing Wang, Pierre Cunin, Samuel Bates, Chevaun Morrison-Smith, Benjamin Nicholson, Edmond Wong, Leena El-Mufti, Michael Kann, Anna Bolling, Brooke Fortin, Hayden Ventresca, Wen Zhou, Santiago Pardo, Megan Kwock, Aditi Hazra, Leo Cheng, Q. Rushdy Ahmad, James A. Toombs, Rebecca Larson, Haley Pleskow, Nell Meosky Luo, Christina Samaha, Unnati M. Pandya, Pushpamali De Silva, Sally Zhou, Zakary Ganhadeiro, Sara Yohannes, Rakiesha Gay, Jacqueline Slavik, Shibani S. Mukerji, Petr Jarolim, David R. Walt, Becky C. Carlyle, Lauren L. Ritterhouse, Sara Suliman

**Affiliations:** 1 Department of Neurology, Massachusetts General Hospital, Harvard Medical School, Charlestown, Boston, MA USA; 2 Department of Medicine, Harvard Medical School, Boston, MA USA; 3 Mass General Brigham Innovation, Boston, MA USA; 4 Cardiology Division, Massachusetts General Hospital, Charlestown, MA USA; 5 Department of Neurosurgery, Brigham and Women’s Hospital, Harvard Medical School, Boston, MA USA; 6 Department of Medical Oncology and Center for Cancer-Genome Discovery, Dana-Farber Cancer Institute, Boston, MA USA; 7 Department of Pathology, Harvard Medical School, Boston, MA USA; 8 Center for Regenerative Medicine, Massachusetts General Hospital, Boston, MA USA; 9 Harvard Stem Cell Institute, Cambridge, MA USA; 10 Department of Psychiatry, Massachusetts General Hospital, Boston, MA USA; 11 Wyss Institute for Biologically Inspired Engineering, Harvard University, Boston, MA USA; 12 Department of Pathology, Brigham and Women’s Hospital, Harvard Medical School, Boston, MA USA; 13 Sackler School of Biomedical Sciences, Tufts University School of Medicine, Boston, MA USA; 14 Division of Rheumatology, Inflammation and Immunity, Brigham and Women’s Hospital, Boston, MA USA; 15 Medical Diagnostic Technology Evaluation, LLC, Carlisle, MA USA; 16 Center for Systems Biology, Massachusetts General Hospital, Boston, MA USA; 17 Department of Pathology, Massachusetts General Hospital, Boston, MA USA; 18 Wellman Center for Photomedicine, Massachusetts General Research Institute, Boston, MA USA; 19 Department of Dermatology, Massachusetts General Hospital, Boston, MA USA; 20 Evergrande Center for Immunologic Diseases, Brigham and Women’s Hospital, Boston, MA USA; 21 Cardiovascular Division, Department of Medicine, Brigham and Women’s Hospital, Boston, MA USA; 22 Functional Genomics Laboratory, Channing Division of Network Medicine, Brigham and Women’s Hospital, Boston, MA USA; 23 Center for Cancer Research, Massachusetts General Hospital, Harvard Medical School, Charlestown, MA USA; 24 Division of Nephrology and Endocrine Unit Department of Medicine, Massachusetts General Hospital, Boston, MA USA; 25 Cancer Center Protocol Office, Massachusetts General Hospital, Boston, MA USA; 26 Division of Preventative Medicine, Brigham and Women’s Hospital, Harvard Medical School, Boston, MA USA; 27 Radiology and pathology, Massachusetts General Hospital, Harvard Medical School, Boston, MA USA; 28 Brigham Research Institute, Brigham and Women’s Hospital, Boston, MA USA; 29 Immunology Program, Harvard Medical School, Boston, MA USA; 30 Cellular Immunotherapy Program, Cancer Center, Massachusetts General Hospital, Boston, MA USA; 31 Department of Radiation Oncology, Massachusetts General Hospital, Harvard Medical School, Boston, MA USA; 32 Folia Health, Inc., Cambridge, MA USA; 33 Vincent Center for Reproductive Biology, Department of Obstetrics and Gynecology, Massachusetts General Hospital, Boston, MA USA; 34 Department of Biology, Northeastern University, Boston, MA USA; 35 College of Science, Northeastern University, Boston, MA USA; 36 Mass General Brigham COVID Center for Innovation, Diagnostics Accelerator, Boston, MA USA

**Keywords:** SARS-CoV-2, Coronavirus, COVID-19, Antibodies, Lateral flow assays

## Abstract

**Background:**

COVID-19 has resulted in significant morbidity and mortality worldwide. Lateral flow assays can detect anti-Severe Acute Respiratory Syndrome Coronavirus-2 (SARS-CoV-2) antibodies to monitor transmission. However, standardized evaluation of their accuracy and tools to aid in interpreting results are needed.

**Methods:**

We evaluated 20 IgG and IgM assays selected from available tests in April 2020. We evaluated the assays’ performance using 56 pre-pandemic negative and 56 SARS-CoV-2-positive plasma samples, collected 10–40 days after symptom onset, confirmed by a molecular test and analyzed by an ultra-sensitive immunoassay. Finally, we developed a user-friendly web app to extrapolate the positive predictive values based on their accuracy and local prevalence.

**Results:**

Combined IgG + IgM sensitivities ranged from 33.9 to 94.6%, while combined specificities ranged from 92.6 to 100%. The highest sensitivities were detected in Lumiquick for IgG (98.2%), BioHit for both IgM (96.4%), and combined IgG + IgM sensitivity (94.6%). Furthermore, 11 LFAs and 8 LFAs showed perfect specificity for IgG and IgM, respectively, with 15 LFAs showing perfect combined IgG + IgM specificity. Lumiquick had the lowest estimated limit-of-detection (LOD) (0.1 μg/mL), followed by a similar LOD of 1.5 μg/mL for CareHealth, Cellex, KHB, and Vivachek.

**Conclusion:**

We provide a public resource of the accuracy of select lateral flow assays with potential for home testing. The cost-effectiveness, scalable manufacturing process, and suitability for self-testing makes LFAs an attractive option for monitoring disease prevalence and assessing vaccine responsiveness. Our web tool provides an easy-to-use interface to demonstrate the impact of prevalence and test accuracy on the positive predictive values.

**Supplementary Information:**

The online version contains supplementary material available at 10.1186/s12879-021-06257-7.

## Background

Coronavirus disease 2019 (COVID-19), caused by infection with the severe acute respiratory syndrome coronavirus 2 (SARS-CoV-2), was declared a global pandemic by on March 11th, 2020 [1], with a second wave of the pandemic well underway [1]. However, accurate estimates of transmission rely on accurate and widely distributed population immunosurveillance tools to measure SARS-CoV-2 infection in diverse community settings. Among SARS-CoV-2-infected individuals, 40–45% are estimated to remain asymptomatic [2], suggesting that prevalence is likely underestimated [3]. Therefore, detecting prior exposure to SARS-CoV-2 as opposed to other viral infections or other coronavirus strains is crucial [4].

There are different types of clinical SARS-CoV-2 tests. Diagnostic testing relies on reverse-transcriptase quantitative polymerase chain reaction (RT-qPCR) and antigen-based immunodiagnostics to detect active infection [5]. Conversely, serological tests are useful for monitoring population prevalence and prior exposure by measuring antibodies against SARS-CoV-2 [6–8]. These include enzyme-linked immunosorbent assays (ELISAs), chemiluminescence assays, and lateral flow assays (LFAs) [5, 9, 10]. LFAs are attractive for home testing and population surveillance, since they are affordable, scalable, rely on easily accessible specimens such as fingerstick whole blood and give a result readout within minutes [7]. Since multiple vaccines received emergency use authorization [11], serological assays could be used to determine whether vaccines elicit a detectable and durable immune response [12–16]. Hence, easy-to-use LFAs will have important applications in the upcoming phases of the pandemic. Since the onset of the COVID-19 epidemic, multiple studies evaluated the accuracy of serological tests [9, 17–21]. Many of these tests received Emergency Use Authorization (EUA) through the Food and Drug Administration (FDA) [22].

Despite the utility of SARS-CoV-2 antibody tests, misinterpretation of results is very likely [23]. A negative serological test result does not preclude prior infection since seroconversion occurs 9–11 and 18–20 days after symptoms onset for IgM and IgG antibodies, respectively [10, 24]. Conversely, positive results do not indicate active infection [23]. Furthermore, the prevalence of SARS-CoV-2 is highly variable [1, 3], and known to directly impact the predictive value of a test result. A higher prevalence increases the likelihood that a positive test result indicates a real infection (i.e. higher positive predictive value) [25], but will also decrease the negative predictive value, resulting in more false negative results [25]. Therefore, accessible tools to assist the public with interpreting results based on test accuracy and different prevalence scenarios are critical [23, 26].

In April 2020, the Mass General and Brigham Center for COVID Innovation direct-to-consumer working group scanned available serological assays and selected 20 lateral flow assays, based on reported assay characteristics and supply chain availability [27]. The LFAs were evaluated by blinded operators using the same samples to standardize the evaluation of their accuracy. Additionally, we developed a user-friendly web-tool to assist the end user to interpret their results. This study provides both the evaluation data to serve as a public resource to guide implementation of LFAs, and the tool to aid the interpretation of home testing results.

## Methods

### Sample procurement

We procured 56 SARS-CoV-2-positive, 46 pre-pandemic SARS-CoV-2-negative, and 10 SARS-CoV-2-negative HIV-positive EDTA plasma samples. SARS-CoV-2 positive EDTA plasma samples were obtained from clinical discards banked within 24–72 h of collection at the Crimson Core of the MGB Biobank, which was composed of hospitalized symptomatic patients. All samples had positive SARS-CoV-2 PCR results using an EUA approved test at Brigham and Women’s Hospital (Panther Fusion SARS-CoV-2 assay, Hologic, Inc., San Diego, CA or Xpert Xpress SARS-CoV-2, Cepheid, Inc., Sunnyvale, CA) or the Clinical Research Sequencing Platform at the Broad Institute of MIT and Harvard (in house Laboratory Developed Test) 10–40 days prior to sample collection. The participants’ charts were reviewed by study staff to identify samples collected ≥10 days after onset of symptoms and to exclude immunosuppressed participants, after which samples were anonymized and stripped of protected health information. Pre-pandemic negative control samples were randomly selected from healthy participants with a Charlson Age-Comorbidity Index [28] score ≤ 2 and EDTA plasma banked in the MGB Biobank between Jan 1-Dec 1, 2019 from inpatients. HIV-positive control samples were obtained from EDTA plasma samples banked prior to January 2020 in a study on neuropathic pain in HIV. All HIV-positive participants were on antiretroviral therapy. For 8 out of the 10 HIV-positive samples, viral load quantification was available and showed 256 copies/ml or less, and 5 showed loads either undetectable or under 20 copies/ml. All samples were collected from consented individuals. Sample and data collection conformed to Good Clinical Practice guidelines and Declaration of Helsinki. The study was approved under the Massachusetts General Brigham (MGB) Institutional Review Board (protocol no. 2020P001204).

### Lateral flow assays (LFAs)

Twenty commercial IgG/IgM lateral flow assays (LFAs) from 18 manufacturers were evaluated (Supplementary Table 1). LFAs were analyzed by blinded operators according to manufacturer instructions for use (IFU), with the exception of using micropipettes instead of manufacturer-provided droppers to minimize technical variability. Samples were thawed on ice, randomized, and brought to room temperature. Kit components were also brought to room temperature. The IFU-specified volumes of sample and buffer were added to the cassette. Specified sample volumes varied for different LFAs but were typically in the 5-20 μL range. The cassettes were run at room temperature on a flat surface and results read immediately after the time interval defined in the IFU (typically ranging from 10 to 15 min). Each cassette was independently scored by two blinded raters as either “positive,” “negative,” or “invalid”. Ratings were designated according to the interpretation guidelines outlined in each individual IFU. Each cassette was photographed under four standardized illumination conditions and viewpoints for future analysis.

### Reproducibility testing

For inter-operator reproducibility analysis, separate pools of EDTA plasma were obtained from > 30 pre-pandemic healthy individuals (negative pool) and > 30 convalescent participants collected after symptom resolution at the Massachusetts General Hospital (MGH) respiratory illness clinic (positive pool). Convalescent samples for the positive pool were confirmed to be positive using the COBAS SARS-CoV-2 PCR test (Roche Diagnostics, Indianapolis, IN) at MGH. A total of 20 replicates per pool were run by two independent pairs of blinded operators, alternating between positive and negative pools (10 replicates per pair). Reproducibility was calculated according to agreement between operator ratings as well as concordance of readout with sample pool COVID status.

### Sensitivity and specificity testing

Our cohort of 112 EDTA plasma samples was used across all 20 LFAs to evaluate performance: sensitivity, specificity, positive predictive value (PPV) and negative predictive value (NPV). Samples were sub-aliquoted throughout the analysis to minimize freeze-thaw cycles. Binary presence/absence classifications were used, and discordant calls were resolved by a third operator inspecting photographs taken of the relevant LFA.

### LFA usability

In addition to initial screening [27], each LFA kit was assessed for consumer usability based on complexity of kit materials, sample requirements, and IFU clarity. Supplied kit components were documented for completeness and examined for ease-of-use. IFU protocols were rated on a scale from 0 to 14 according to a predefined rubric (Supplementary Table 2) by three independent raters. Usability evaluations are shown in Supplementary Table 3. Sample input requirements for each LFA are in Supplementary Table 1.

### Ultrasensitive Simoa serology assays

Plasma samples were diluted 4000-fold, and the total IgG and IgM levels against the SARS-CoV-2 spike protein were measured using a custom Single Molecule Array (Simoa) assay as described [29] on an automated HD-X Analyzer (Quanterix, Billerica, MA, USA), to provide a quantitative reference for anti-spike antibody titers in the plasma samples. Normalized mean Average Enzymes per Bead (AEB) levels were calculated using a standard set of calibrators produced by serially diluting a large volume of plasma from seroconverted individuals. Antibody concentrations were estimated using a calibration curve of recombinant anti-SARS-CoV-2 antibodies [30].

#### Analysis and Webapp

Detailed methods are in the supplementary.

## Results

### Study population

We obtained plasma samples from 56 pre-pandemic patients, including 10 HIV+, and 56 symptomatic inpatients in March and April 2020. HIV-negative samples were matched for sex and age. The overall study population included 25.9% Blacks, 4.5% Latinx, 9.8% Asians, and 43.8% Non-Hispanic Whites (Table 1). COVID+ samples were from individuals between 10 and 40 days post symptom onset, with 60.7% samples taken between 2 and 4 weeks. Of all the COVID+ participants, 7 (12.5%) were symptomatic outpatients and 49 (87.5%) were hospitalized. Among those hospitalized, 25 (51%) required intensive care unit (ICU) treatment and 4 (8.2%) were deceased at the time of chart review (Supplementary Table 4). Additional mortalities were possible after chart review since some participants were in critical condition in the ICU.

**Table 1:** Demographic information of all individuals whose plasma samples were used for this study. Individuals are broken out by COVID+/− and HIV+/− status. Black includes one mixed African American in the COVID-HIV+ group

	COVID- HIV-	COVID- HIV+	COVID+ HIV-	Overall
	*n* = 46	*n* = 10	*n* = 56	*n* = 112
**Sex**				
Female	28 (60.9%)	1 (10%)	31 (55.4%)	60
Male	18 (39.1%)	9 (90%)	25 (44.6%)	52
**Age**				
Mean (SD)	46.7 (13.4)	58.6 (5.3)	58.7 (20.4)	53.8 (17.8)
Median [Min, Max]	48.0 [19.0, 68.0]	58.0 [48.0,65.0]	57.5 [24.0, 98.0]	54.5 [19.0,98.0]
**Race**				
Asian	8 (17.4%)	0 (0%)	3 (5.4%)	11 (9.8%)
Black	10 (21.7%)	4 (40%)	16 (28.6%)	30 (26.8%)
LatinX	0 (0%)	0 (0%)	5 (8.9%)	5 (4.5%)
Unknown/Other	8 (17.4%)	1 (10%)	8 (14.3%)	17 (15.2%)
White	20 (43.5%)	5 (50%)	24 (42.9%)	49 (43.8%)

### LFA performance: reproducibility

**Fig. 1:**
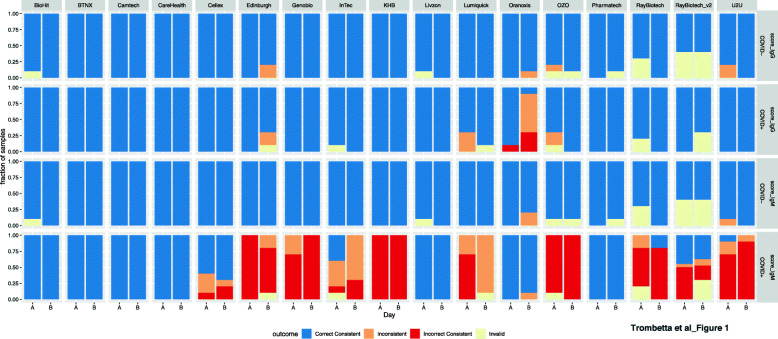
LFA reproducibility. Colors represent LFA outcome performed across two days (A & B) by two independent pairs of operators on a COVID positive (COVID+) pooled sample and a COVID negative (COVID-) pooled sample. On each day ten technical replicates of each pool were performed. Blue represents replicates where both operators that day agreed on the outcome, and the outcome was correctly called positive or negative. Red represents occasions where both operators agreed, but the outcome was incorrectly called. Orange represents replicates where the operators did not agree on the LFA outcome. Light yellow represents an invalid test where control bands did not meet criteria for a valid test. IgG was the most reproducible isotype for all LFAs except Oranoxis, and consistent false negatives were common occurrences for IgM for the majority of LFAs

Four independent operators working in teams of two on separate days applied each pool 10 times to each LFA, with processing as dictated by the instructions for use (IFU). API version1 LFAs were not assessed for reproducibility due to limited cassette availability and the high volume required. Of the remaining 19 LFAs, three (BTNX, Camtech, and Carehealth) had 100% consistent correct outcomes across both isotypes, with an additional three (BioHit, Zhuhai Livzon, and Phamatech) having no incorrect or inconsistent outcomes with one or two invalid tests (Fig. 1). IgG was the more reproducible isotype. The majority of incorrect consistent calls came from operators calling a COVID+ sample IgM negative (Fig. 1).

### LFA performance: practicalities of use

We assessed the LFAs according to this rubric (Supplementary Table 2) by three independent raters and assigned a composite score on a scale of 0–14 (Supplementary Table 3). Five LFAs (BioHit, InTec, Lumiquick, Phamatech, and U2U) received full scores for IFU clarity. LFAs frequently lost points for imprecise instructions regarding time between adding sample and having sufficient sample flow to read the results. Important kit usability criteria, such as whether the included pipette droppers show clear volume markings, were also recorded (Supplementary Table 3). The IFU ambiguity led to administration of too much or little sample for a valid test. While these IFUs may not yet be intended for the general public, it will be important to clarify the instructions moving forward and include droppers to minimize potential for sample volume errors.

### LFA performance: sensitivity and specificity

To focus on LFA specificity, given the likely use of these tests in low-prevalence settings, disagreement between operators was interpreted as a negative call. Across all but three of the LFAs (Biohit, BTNX, and Vivacheck), sensitivity was higher for IgG than IgM. Sensitivity for IgG ranged from 98.2% (Lumiquick) down to 72.7% (Oranoxis), and for IgM from 96.4% (BioHit) to 23.2% (Oxo and U2U) (Table 2). LFA specificity was much higher for both isotypes, with 11 LFAs having a specificity of 100% for IgG (API, API v2, BTNX, Camtech, Genobio, Oranoxis, Phamatech, Ray Biotech, Ray Biotech v2, U2U, and Zhuhai Livzon), and 8 LFAs having a specificity of 100% for IgM (API, CareHealth, Cellex, Lumiquick, Oranoxis, Ray Biotech v2, U2U, and Zhuhai Livzon) (Table 2). Under the assumption that the likelihood of two randomly occurring false positives for any one individual is low, an IgG/IgM composite score (averaged operator scores, see Supplementary Methods) was produced to maximize test specificity. Using this composite score, all LFAs except BioHit, Cellex, Edinburgh, InTec, and Vivachek achieved a specificity of 100%. This result underscores the potential of considering the outcome in both isotypes to minimize false positives, although it is more likely that a single isotype will be used in clinical testing.

**Table 2:** LFA performance in terms of sensitivity and specificity

**IgG lateral flow assay performance**	Manufacturer	N	TP	TN	FP	FN	sensitivity	specificity	PPV	NPV	sensitivity_CI95	specificity_CI95	PPV_CI95	NPV_CI95
	API	112	52	56	0	4	0.929	1	1	0.933	(0.853–1)	(0.991–1)	(0.99–1)	(0.861–1)
	API (v2)	112	52	56	0	4	0.929	1	1	0.933	(0.853–1)	(0.991–1)	(0.99–1)	(0.861–1)
	BioHit	110	52	50	4	4	0.929	0.926	0.929	0.926	(0.853–1)	(0.847–1)	(0.853–1)	(0.847–1)
	BTNX	110	48	56	0	6	0.889	1	1	0.903	(0.796–0.982)	(0.991–1)	(0.99–1)	(0.821–0.985)
	Camtech	111	48	55	0	8	0.857	1	1	0.873	(0.756–0.958)	(0.991–1)	(0.99–1)	(0.783–0.963)
	CareHealth	112	53	53	3	3	0.946	0.946	0.946	0.946	(0.878–1)	(0.878–1)	(0.878–1)	(0.878–1)
	Cellex	112	53	52	4	3	0.946	0.929	0.93	0.945	(0.878–1)	(0.853–1)	(0.855–1)	(0.876–1)
	Edinburgh	108	47	51	2	8	0.855	0.962	0.959	0.864	(0.753–0.957)	(0.901–1)	(0.893–1)	(0.768–0.96)
	Genobio	111	42	56	0	13	0.764	1	1	0.812	(0.643–0.885)	(0.991–1)	(0.988–1)	(0.713–0.911)
	InTec	111	49	54	1	7	0.875	0.982	0.98	0.885	(0.779–0.971)	(0.938–1)	(0.931–1)	(0.797–0.973)
	KHB	112	53	54	2	3	0.946	0.964	0.964	0.947	(0.878–1)	(0.906–1)	(0.906–1)	(0.88–1)
	Lumiquick	111	54	53	3	1	0.982	0.946	0.947	0.981	(0.938–1)	(0.878–1)	(0.88–1)	(0.935–1)
	Oranoxis	110	40	55	0	15	0.727	1	1	0.786	(0.6–0.854)	(0.991–1)	(0.988–1)	(0.683–0.889)
	OZO	112	43	54	2	13	0.768	0.964	0.956	0.806	(0.649–0.887)	(0.906–1)	(0.885–1)	(0.704–0.908)
	Phamatech	95	38	47	0	10	0.792	1	1	0.825	(0.667–0.917)	(0.989–1)	(0.987–1)	(0.718–0.932)
	Ray Biotech	112	43	56	0	13	0.768	1	1	0.812	(0.649–0.887)	(0.991–1)	(0.988–1)	(0.713–0.911)
	Ray Biotech (v2)	111	51	55	0	5	0.911	1	1	0.917	(0.827–0.995)	(0.991–1)	(0.99–1)	(0.839–0.995)
	U2U	112	43	56	0	13	0.768	1	1	0.812	(0.649–0.887)	(0.991–1)	(0.988–1)	(0.713–0.911)
	Vivachek	111	52	55	1	3	0.945	0.982	0.981	0.948	(0.876–1)	(0.938–1)	(0.935–1)	(0.882–1)
	Zhuhai Livzon	109	47	54	0	8	0.855	1	1	0.871	(0.753–0.957)	(0.991–1)	(0.989–1)	(0.779–0.963)
**IgM lateral flow assay performance**	Manufacturer	N	TP	TN	FP	FN	sensitivity	specificity	PPV	NPV	sensitivity_CI95	specificity_CI95	PPV_CI95	NPV_CI95
	API	112	27	56	0	29	0.482	1	1	0.659	(0.342–0.622)	(0.991–1)	(0.981–1)	(0.552–0.766)
	API (v2)	112	51	55	1	5	0.911	0.982	0.981	0.917	(0.827–0.995)	(0.938–1)	(0.934–1)	(0.839–0.995)
	BioHit	110	54	49	5	2	0.964	0.907	0.915	0.961	(0.906–1)	(0.82–0.994)	(0.835–0.995)	(0.898–1)
	BTNX	110	48	53	3	6	0.889	0.946	0.941	0.898	(0.796–0.982)	(0.878–1)	(0.867–1)	(0.812–0.984)
	Camtech	111	42	52	3	14	0.75	0.945	0.933	0.788	(0.628–0.872)	(0.876–1)	(0.849–1)	(0.682–0.894)
	CareHealth	112	50	56	0	6	0.893	1	1	0.903	(0.803–0.983)	(0.991–1)	(0.99–1)	(0.821–0.985)
	Cellex	112	31	56	0	25	0.554	1	1	0.691	(0.415–0.693)	(0.991–1)	(0.984–1)	(0.584–0.798)
	Edinburgh	108	29	49	4	26	0.527	0.925	0.879	0.653	(0.386–0.668)	(0.845–1)	(0.753–1)	(0.539–0.767)
	Genobio	111	31	52	4	24	0.564	0.929	0.886	0.684	(0.424–0.704)	(0.853–1)	(0.766–1)	(0.573–0.795)
	InTec	111	43	54	1	13	0.768	0.982	0.977	0.806	(0.649–0.887)	(0.938–1)	(0.921–1)	(0.704–0.908)
	KHB	112	31	55	1	25	0.554	0.982	0.969	0.688	(0.415–0.693)	(0.938–1)	(0.893–1)	(0.58–0.796)
	Lumiquick	111	37	56	0	18	0.673	1	1	0.757	(0.54–0.806)	(0.991–1)	(0.986–1)	(0.653–0.861)
	Oranoxis	110	48	55	0	7	0.873	1	1	0.887	(0.776–0.97)	(0.991–1)	(0.99–1)	(0.8–0.974)
	OZO	112	13	54	2	43	0.232	0.964	0.867	0.557	(0.113–0.351)	(0.906–1)	(0.662–1)	(0.453–0.661)
	Phamatech	95	38	46	1	10	0.792	0.979	0.974	0.821	(0.667–0.917)	(0.927–1)	(0.911–1)	(0.712–0.93)
	Ray Biotech	112	40	55	1	16	0.714	0.982	0.976	0.775	(0.587–0.841)	(0.938–1)	(0.917–1)	(0.671–0.879)
	Ray Biotech (v2)	111	47	55	0	9	0.839	1	1	0.859	(0.734–0.944)	(0.991–1)	(0.989–1)	(0.766–0.952)
	U2U	112	13	56	0	43	0.232	1	1	0.566	(0.113–0.351)	(0.991–1)	(0.962–1)	(0.463–0.669)
	Vivachek	111	51	55	1	4	0.927	0.982	0.981	0.932	(0.849–1)	(0.938–1)	(0.934–1)	(0.859–1)
	Zhuhai Livzon	109	46	54	0	9	0.836	1	1	0.857	(0.729–0.943)	(0.991–1)	(0.989–1)	(0.763–0.951)
**Combined IgG + IgM performance**	Manufacturer	N	TP	TN	FP	FN	sensitivity	specificity	PPV	NPV	sensitivity_CI95	specificity_CI95	PPV_CI95	NPV_CI95
	API	112	36	56	0	20	0.643	1	1	0.737	(0.509–0.777)	(0.991–1)	(0.986–1)	(0.631–0.843)
	API (v2)	112	51	56	0	5	0.911	1	1	0.918	(0.827–0.995)	(0.991–1)	(0.99–1)	(0.841–0.995)
	BioHit	110	53	50	4	3	0.946	0.926	0.93	0.943	(0.878–1)	(0.847–1)	(0.855–1)	(0.871–1)
	BTNX	110	47	56	0	7	0.87	1	1	0.889	(0.771–0.969)	(0.991–1)	(0.989–1)	(0.803–0.975)
	Camtech	111	44	55	0	12	0.786	1	1	0.821	(0.67–0.902)	(0.991–1)	(0.989–1)	(0.722–0.92)
	CareHealth	112	51	56	0	5	0.911	1	1	0.918	(0.827–0.995)	(0.991–1)	(0.99–1)	(0.841–0.995)
	Cellex	112	46	55	1	10	0.821	0.982	0.979	0.846	(0.712–0.93)	(0.938–1)	(0.927–1)	(0.751–0.941)
	Edinburgh	108	35	51	2	20	0.636	0.962	0.946	0.718	(0.5–0.772)	(0.901–1)	(0.86–1)	(0.606–0.83)
	Genobio	111	39	56	0	16	0.709	1	1	0.778	(0.58–0.838)	(0.991–1)	(0.987–1)	(0.675–0.881)
	InTec	111	46	54	1	10	0.821	0.982	0.979	0.844	(0.712–0.93)	(0.938–1)	(0.927–1)	(0.747–0.941)
	KHB	112	39	56	0	17	0.696	1	1	0.767	(0.567–0.825)	(0.991–1)	(0.987–1)	(0.663–0.871)
	Lumiquick	111	40	56	0	15	0.727	1	1	0.789	(0.6–0.854)	(0.991–1)	(0.988–1)	(0.687–0.891)
	Oranoxis	110	43	55	0	12	0.782	1	1	0.821	(0.664–0.9)	(0.991–1)	(0.988–1)	(0.722–0.92)
	OZO	112	19	56	0	37	0.339	1	1	0.602	(0.206–0.472)	(0.991–1)	(0.974–1)	(0.497–0.707)
	Phamatech	95	36	47	0	12	0.75	1	1	0.797	(0.617–0.883)	(0.989–1)	(0.986–1)	(0.686–0.908)
	Ray Biotech	112	41	56	0	15	0.732	1	1	0.789	(0.607–0.857)	(0.991–1)	(0.988–1)	(0.687–0.891)
	Ray Biotech (v2)	111	48	55	0	8	0.857	1	1	0.873	(0.756–0.958)	(0.991–1)	(0.99–1)	(0.783–0.963)
	U2U	112	22	56	0	34	0.393	1	1	0.622	(0.256–0.53)	(0.991–1)	(0.977–1)	(0.516–0.728)
	Vivachek	111	52	55	1	3	0.945	0.982	0.981	0.948	(0.876–1)	(0.938–1)	(0.935–1)	(0.882–1)
	Zhuhai Livzon	109	45	54	0	10	0.818	1	1	0.844	(0.707–0.929)	(0.991–1)	(0.989–1)	(0.747–0.941)

*N* Total number of valid assays; *TP* True Positive; *TN* True Negative; *FP* False Positive; *FN* False Negative; *PPV* Positive Predictive Value; *NPV* Negative Predictive Value; *CI95*: 95% confidence interval

**Fig. 2:**
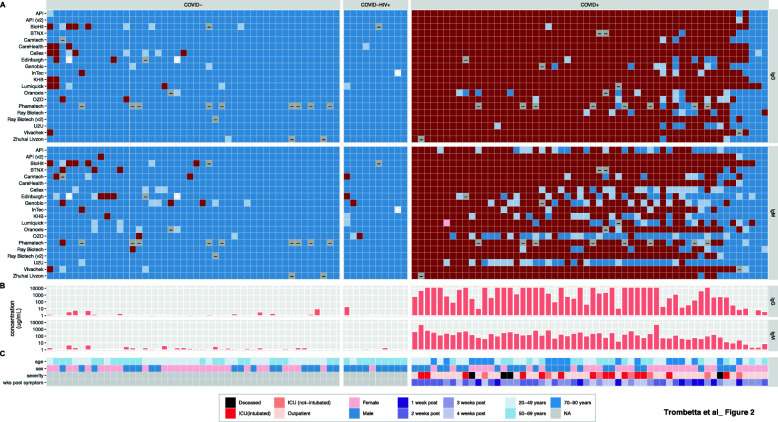
Per-individual LFA performance. Colors represent scores computed for IgG (top panel) and IgM (lower panel), where dark red = + 1 (operators all agree a band is present) and dark blue = −1 (operators all agree there is no band) with intermediate colors (pink and light blue) representing varying degrees of operator disagreement. Grey represents invalid runs. Samples are ordered within the COVID-, COVID-HIV+, and COVID+ groups in order of decreasing average score across all LFAs for both antibodies. Clinical variables include age (< 50 yrs.: light blue, 50-69 yrs.: intermediate blue, ≥70 yrs.: dark blue), sex (blue: male, pink: female), disease severity (hospitalized: light green, ICU: orange, ICU + respirator: red, deceased: black), and weeks post symptom-onset (dark green: 1–2 weeks, lightest green: ≥5 weeks)

We created heatmaps to visualize individual sample outcomes across all LFAs to assess whether we systematically detected the same miscalls across multiple LFAs (Fig. 2). False negatives (blue squares in the COVID+ panel) amongst COVID+ patients were somewhat reproducible, with three COVID+ samples called negative in both isotypes by all, or all but one LFA. These miscalls were not clearly explained by known demographics (age, sex) or clinical variables (disease severity, weeks post symptom onset) (Fig. 2, bottom panel). To investigate whether these miscalls were related to low titers of anti-SARS-CoV-2 antibodies from participants with a suppressed immune response, all 112 samples were analyzed for anti-spike IgG and IgM antibodies using a custom quantitative Simoa assay [29]. The three samples that were called negative across almost all LFAs, which were also 2–3 weeks post-symptoms, had the lowest levels of anti-spike antibodies in COVID+ samples for both IgG and IgM, suggesting these participants had a slower or suppressed immune response to SARS-CoV-2 infection.

Unlike false negatives, most false positives (Fig. 2, red squares in COVID- panel) amongst COVID- individuals appear largely uncorrelated between LFAs. However, two samples showed IgG false positives across multiple LFAs, which may suggest long lasting antibodies from exposure to coronaviruses other than SARS-CoV-2. Nonetheless, this observation is not reflected in the antibody levels from the Simoa analysis, which showed barely detectable anti-spike antibodies in these two samples.

### Defining the limit of detection for qualitative LFAs

**Fig. 3:**
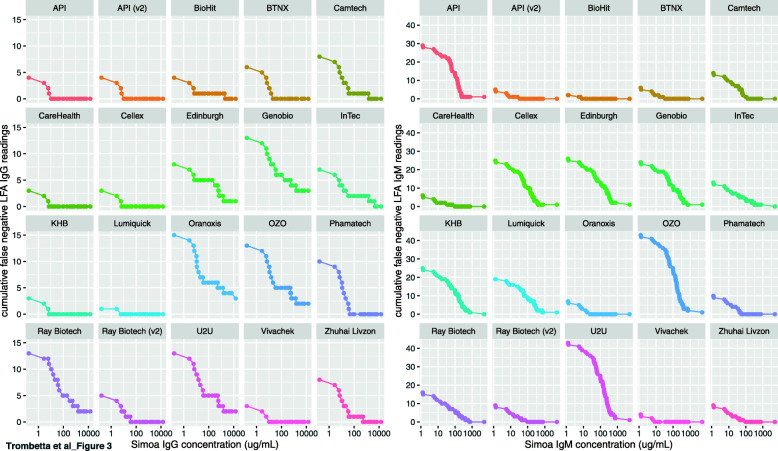
Determining the limit of detection of qualitative LFAs. Samples were ranked from highest concentration of anti-spike antibody (determined by Simoa) on the right, to lowest concentration (x-axis). As the sample concentration decreases to the left, a cumulative count of false negatives is shown on the y-axis. IgG is shown on the left, IgM on the right. Note the difference in magnitude of the y-axes between IgG and IgM; these LFAs are generally more sensitive to IgG than IgM

The custom Simoa anti-spike IgG and IgM antibody assays use a standard curve to determine standardized antibody concentration in each sample [29]. The results obtained from this assay can therefore be used to estimate a limit of detection (LOD) for each of the qualitative LFAs. The cumulative number of false negative LFA calls in COVID+ samples (Fig. 3, y-axis) were computed as a function of decreasing antibody concentrations (Fig. 3, x-axis) separately for IgG and IgM. We define the LOD for each LFA/antibody as the concentration at which ≥95% of the COVID+ samples are called unambiguously positive (Supplementary Table 5). Using this definition, for IgG all LFAs (except Genobio, Oranoxis, OZO, Ray Biotech, and U2U) have an LOD within the linear range of the SIMOA assay (1–10,000 μg/mL). Lumiquick has the lowest LOD at 0.1 μg/mL, which was extrapolated by dilution to be within the linear range of the SIMOA standards, and CareHealth, Cellex, KHB, and Vivachek all have an LOD of 1.5 μg/mL (Supplementary Table 5). Considering the generally lower sensitivity observed with the IgM assays (Fig. 2), IgM assays consistently have higher LODs, with 9 exceeding 1000μg/mL. BioHit has the lowest IgM LOD at 0.6 μg/mL, and API v2, BTNX, CareHealth, and Vivachek all have LODs under 10 μg/mL.

### Interpreting test positivity with low prevalence in the general population

**Fig. 4:**
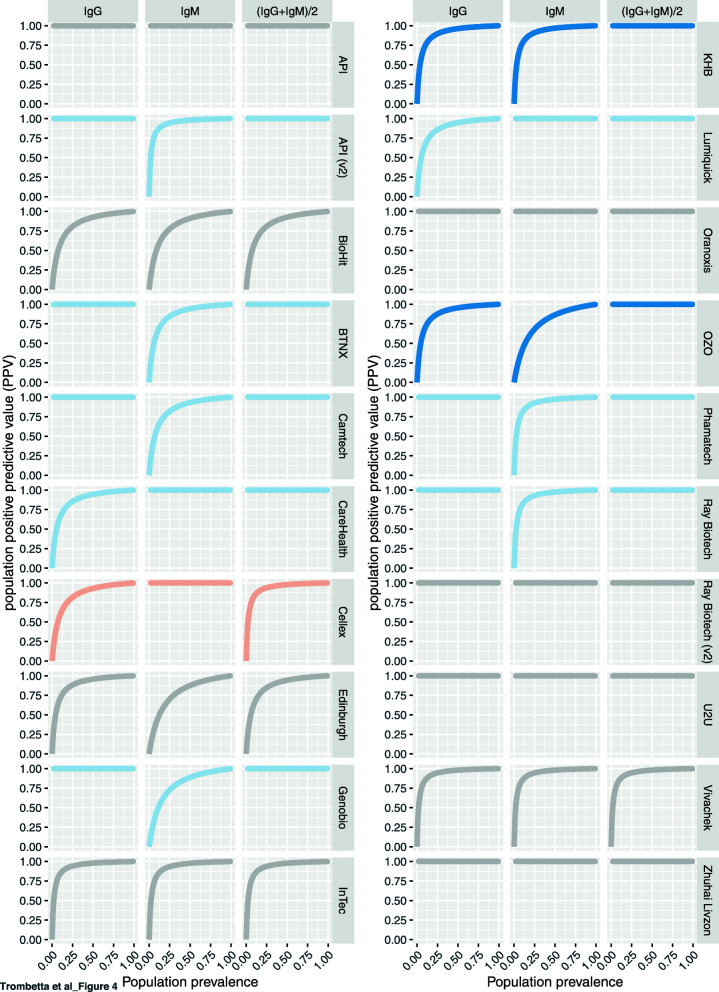
Effect of a changing disease prevalence (x axis) on the population-level PPV of each test for scores derived from IgG (left), IgM (middle), and the average score of both antibodies (right). As disease prevalence increases, the high burden on specificity of LFAs is reduced. Color coding: Grey: no improvement; Light blue: rescues one of the two antibodies; Dark blue: rescues both of the antibodies; Orange: performs worse than one of the antibodies

Low prevalence places a high burden on specificity [25]. Given the high proportion of true negative individuals in the population being studied, prevalence increases the ratio of false positive to true positive test outcomes [31]. Positive predictive values (PPV) correspond to the likelihood that a positive test result reflect true positivity as measured by a gold standard PCR result. We computed PPVs as a function of the fraction of the population infected with (and assumed to have produced antibodies to) SARS-CoV-2 (Fig. 4). Here we see that even with the conservative interpretation of LFA outcome (requiring the majority of operators to see a band to call a sample positive for either IgG or IgM), when the population seroprevalence is ~ 5% the PPV for these assays spans a large range (from ~ 30 to 100%). As the population prevalence increases, the burden on specificity is decreased, and at 50% prevalence the PPV of all LFAs is above 87.5%. Posterior PPV can be improved for most LFAs by requiring both IgG and IgM to be read as positive in order to count an individual as positive (Fig. 4, right panels).

**Fig. 5:**
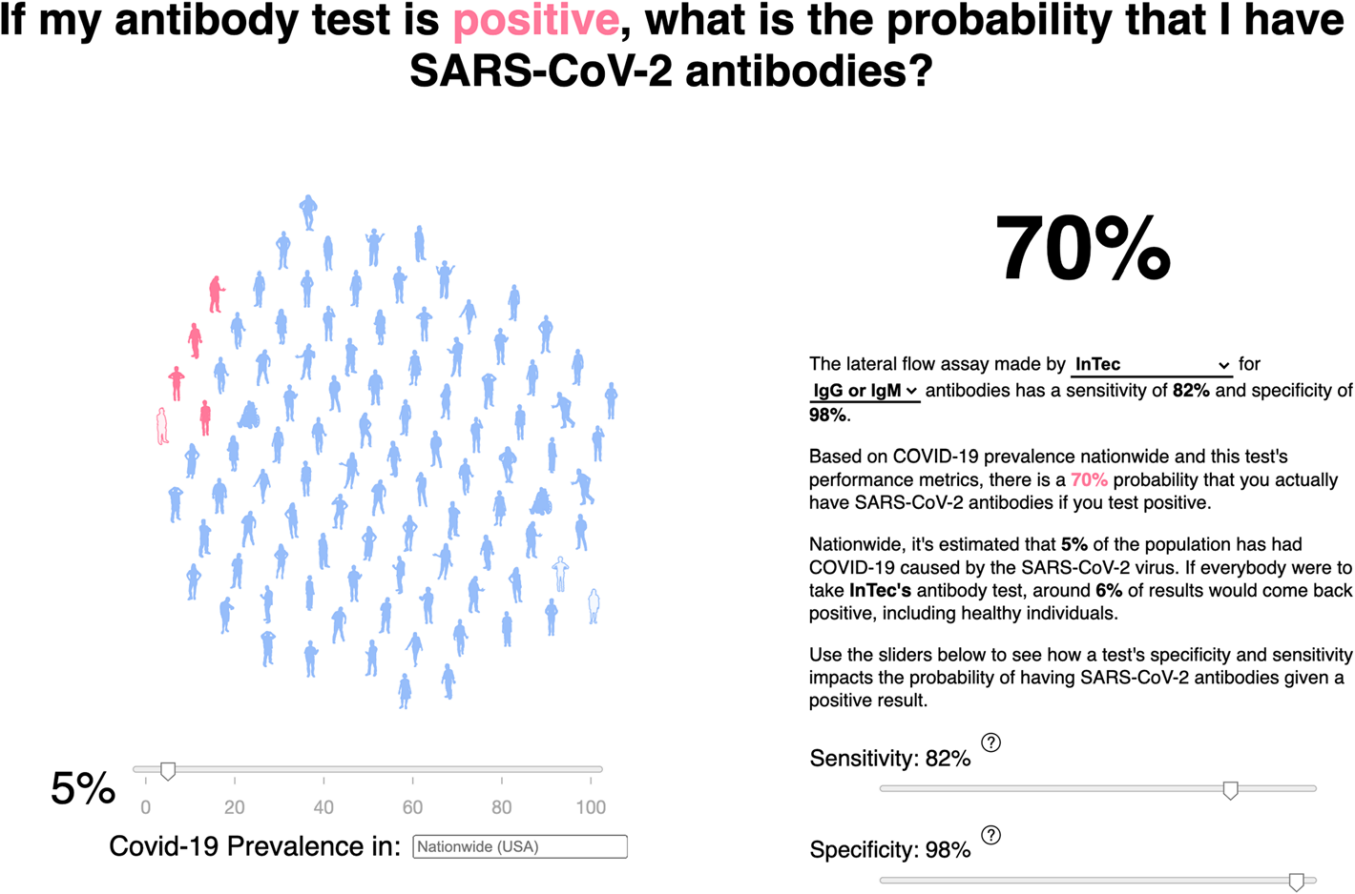
Example image of the interactive webapp hosted at https://covid.omics.kitchen. Using the sliders, one is able to visualize the effect of changing the disease prevalence (and the test performance) on the resulting probability that, given a positive LFA test, the individual does in fact have SARS-CoV-2 antibodies. The figure shows an example of positive predictive value using the US national prevalence of 5% (on November 27th, 2020) and the performance characteristics of the InTec LFA

To visualize the effect of changing population prevalence on the PPV, we created an interactive webapp (https://covid.omics.kitchen; Fig. 5), which allows the user to extrapolate the likelihood that they do in fact have SARS-CoV-2 antibodies if they have a positive LFA result, given the infection prevalence and test accuracy. The app includes benchmark performance of all 20 LFAs evaluated here, as well as those reported in Whitman et al. [10] (filtered to remove samples taken under 10 days post symptom-onset). In an effort to further generalize the utility of this tool, we allow the user to explore the effects of assay performance under difference prevalence scenarios, and to input the reported prevalence data from specific geographic locations within the US [1], based on national, state, and county records.

## Discussion

In this study, we report a standardized cross-evaluation of LFAs on the same pre-pandemic SARS-CoV-2-negative and PCR-confirmed SARS-CoV-2-positive samples, and rate their reproducibility, usability, and performance characteristics. Overall, the LFAs showed a higher propensity for false negative than false positive readings. Results are public: https://covidinnovation.partners.org/evaluation/. We use the Simoa technology [29] to measure the concentrations of anti-spike protein IgG and IgM antibodies and extrapolate the assay limit of detection. We also established a web tool to aid users in understanding the likelihood they have anti-SARS-CoV-2 antibodies given a positive test result. This resource of performance characteristics of several LFAs and a tool for result interpretation, can both be used for immunosurveillance and future home testing applications.

LFAs are tractable tools to estimate community seroprevalence, especially with anticipated seasonal fluctuations in the transmission dynamics of SARS-CoV-2 and other viruses that cause the common cold, which confound the symptomatologic diagnosis of COVID-19 [32]. As new waves of the COVID-19 pandemic resurge around the globe [1], and with commencing vaccinations against SARS-CoV-2 infections [12–14, 16], there is a renewed interest in serological tests to detect anti-SARS-CoV-2 antibodies [20, 33]. Affordable LFAs offer an attractive option for monitoring the presence and longevity of anti-SARS-CoV-2 antibodies, and determining population-level herd immunity [34]. LFAs also obviate the need for complex laboratories to process the samples [35]. As the pandemic expands to previously unexposed communities, it is critical to use simple tools to monitor exposure dynamics and seroconversion in SARS-CoV-2-exposed individuals, as well as vaccine-induced immunity [16]. We tested a mixture of LFAs targeting SARS-CoV-2 nucleocapsid, spike proteins, or both. Moderna’s mRNA-1273 and Pfizer-BioNTech COVID-19 vaccines encode SARS-CoV-2 spike proteins to induce anti-spike antibodies [14, 36]. Therefore, LFAs targeting the spike and nucleocapsid proteins of SARS-CoV-2 could be used to differentiate vaccine- and infection-induced antibody responses, respectively.

Rigorous evaluation of these LFAs by manufacturer-independent parties is important. The US FDA independently reviews medical products before commercialization. The FDA used Emergency Use Authorization (EUA) authority to accelerate the implementation of diagnostic products during the pandemic. Commercial manufacturers were required to submit a completed EUA request [22]. Unfortunately, the rush to market introduced many tests that did not meet typical US or international standards [37]. Therefore, the FDA and international regulatory agencies continue to update guidelines for authorization of new serological tests. Our evaluation plan mirrored the FDA guidelines for evaluating serological tests [22]. We included 10 HIV-positive samples to test whether they have higher false positive results in SARS-CoV-2-negative samples [38], and did not detect higher false positive results.

The mere detection of IgG or IgM responses does not guarantee that neutralizing antibodies are present at protective titers [7, 39]. The study demographics suggest a slight over-representation of African Americans among cases, as reported [40]. However, the sample size was underpowered to formally determine the effect of race on test performance. In our analysis, IgM detectability was less sensitive and reproducible than IgGs across multiple LFAs, possibly due to both lower IgM titres, and lower limits of detection for the IgM LFAs. Waning antibody responses have been reported in some SARS-CoV-2-infected individuals [41–44]. Furthermore, reported cases of re-infection with SARS-CoV-2 suggest that prior exposure, and even seroconversion, do not universally protect against SARS-CoV-2 infection [44, 45]. This could result from low antibody titers as shown in an immunocompromised patient [46], low durability of infection-induced antibodies [42–44], or low neutralizing potential of SARS-CoV-2 infection-induced antibodies in some individuals [34]. IgG and IgM antibodies may also target irrelevant epitopes outside the spike and receptor binding domains, and consequently be less efficient at intercepting infection by the virus [47]. The WHO cautions against interpreting presence of antibodies, even neutralizing ones, as lower risk of re-infection and transmission [48]. The presence of antibodies could be however used for rapid immunosurveillance to monitor extent of population transmission, particularly in asymptomatic but SARS-CoV-2-exposed individuals [3, 33].

One major concern about the deployment of these tests is the misinterpretation of positive results [7, 39]. As more tests move towards FDA approval of home use, clear scientific communication about the result interpretation becomes more crucial [23, 39]. A positive serological result does not necessarily mean active infection [23, 31, 49]. Although combined use of molecular and seroconversion results can be used to confirm the diagnosis of symptomatic and hospitalized individuals [35], a positive serological test in the absence of symptoms dissociates the presence of the antibodies from the time of infection [44]. Additionally, it is important to understand the implication of false positive and false negative results, particularly in the context of a low to mid-prevalence disease such as COVID-19 [25]. Low prevalence decreases the negative predictive value of a test, but increases the false positive rates [25]. A false positive serological test result may prematurely instill confidence that one has immunity against SARS-CoV-2 infection, thus resulting in behavioral changes that increase risk of transmission [50]. Hence, the probability that a person without antibodies will test negative on a serological test is more important than test sensitivity [48–50].

Our study presents a few limitations. Although we successfully benchmarked the performance of the LFAs to a quantitative assay [29], we did not determine the neutralizing potential of these antibodies. Secondly, samples were acquired when PCR testing was restricted to severely ill patients. For epidemiological studies and population surveillance, it will be important to evaluate assay performance on asymptomatic individuals.

## Conclusions

Our study provides a public resource to aid researchers, healthcare providers, public health professionals, and industries impacted by the pandemic such as airlines, in choosing the appropriate serological LFAs for their intended use cases.

## Supplementary Information

**Additional file 1: Supplementary Methods. Supplementary Table 1.** LFA commercial kit information, sample requirements, and protocol details. **Supplementary Table 2.** IFU clarity rubric. **Supplementary Table 3.** Usability ratings and description of kit components. **Supplementary Table 4.** Clinical information for the COVID-positive individuals whose plasma was used in this study. **Supplementary Table 5.** LFA Limits of detection estimated from anti-spike antibodies concentrations measured by Simoa.

## Data Availability

Evaluation data are available at: https://covidinnovation.partners.org/evaluation/. Web application is available at: https://covid.omics.kitchen/

## Abbreviations

AEB: Average Enzymes per Beads
COVID-19: Coronavirus Disease of 2019
EDTA: Ethylenediaminetetraacetic acid
ELISA: Enzyme-linked immunosorbent assay
EUA: Emergency Use Authorization
FDA: Food and Drug Administration
HIV: Human Immunodeficiency Virus
ICU: Intensive Care Units
IFU: Instructions-For-Use
IgG: Immunoglobulin G
IgM: Immunoglobulin M
LFA: Lateral Flow Assay
LOD: Limit of Detection
MGB: Mass General Brigham
MGH: Mass General Hospital
NPV: Negative Predictive Value
PPV: Positive Predictive Value
RT-qPCR: Reverse Transcriptase Quantitative Polymerase Chain Reaction
SARS-CoV-2: Severe Acute Respiratory Syndrome Coronavirus-2
Simoa: Single Molecule Array
