# Supplementary material for: Evaluation of serological lateral flow assays for severe acute respiratory syndrome coronavirus-2
Source: BMC Infect Dis. 2021 Jun 16;21:580. doi: 10.1186/s12879-021-06257-7 (PMC8206878; doi:10.1186/s12879-021-06257-7)
Supplement: Supplementary file 1 — Additional file 1: Supplementary Methods. Supplementary Table 1. LFA commercial kit information, sample requirements, and protocol details. Supplementary Table 2. IFU clarity rubric. Supplementary Table 3. Usability ratings and description of kit components. Supplementary Table 4. Clinical information for the COVID-positive individuals whose plasma was used in this study. Supplementary Table 5. LFA Limits of detection estimated from anti-spike antibodies concentrations measured by Simoa. [file 12879_2021_6257_MOESM1_ESM.pdf]

Title: Evaluation of serological lateral flow assays for severe acute respiratory syndrome coronavirus-2

Author list:

Bianca A. Trombetta[1*], Savannah E. Kandigian[1*], Robert R. Kitchen[2,3*], Korneel Grauwet[4*], Pia Kivisäkk Webb[1,2], Glenn A. Miller[3], Charles G. Jennings[4,5], Sejal Jain[6,7], Samara Miller[2,8,9,10], Yikai Kuo[1,4], Thadryan Sweeney[4], Tal Gilboa[11,12], Maia Norman[11,12,13], Daimon P. Simmons[14], Christopher E. Ramirez[1], Melissa Bedard[14], Catherine Fink[15], Jina Ko[10,16], Esmarline J. De León Peralta[17,18,19], Gerald Watts[14], Emma Gomez-Rivas[14], Vannessa Davis[12], Rocky Barilla[20], Jianing Wang[21], Pierre Cunin[14], Samuel Bates[22], Chevaun Morrison-Smith[12], Benjamin Nicholson[23], Edmond Wong[23], Leena El-Mufti[1], Michael Kann[23], Anna Bolling[1], Brooke Fortin[1], Hayden Ventresca[21], Wen Zhou[24], Santiago Pardo[1], Megan Kwock[25], Aditi Hazra[2,26], Leo Cheng[27], Q. Rushdy Ahmad[11], James A. Toombs[28], Rebecca Larson[29,30], Haley Pleskow[23,31], Nell Meosky Luo[31], Christina Samaha[31], Unnati M. Pandya[2,33], Pushpamali De Silva[18], Sally Zhou[34,35], Zakary Ganhadeiro[34,35], Sara Yohannes[28], Rakeisha Gay[28, 35], Jacqueline Slavik[28], Shibani Mukerji[1], Petr Jarolim[7,12], David R. Walt[11,12], Becky C. Carlyle[1,2], Lauren L. Ritterhouse[17**], Sara Suliman[14**#]

* and ** contributed equally

#Corresponding Author:

Sara Suliman, MPH, PhD, Division of Rheumatology, Inflammation and Immunity, Brigham and Women's Hospital, Boston MA. Diagnostics Accelerator Working Group and Laboratory of the Mass General and Brigham Center for COVID Innovation (MGBCCI), 149 Navy Yard room: 4325, Charlestown MA, USA. Email: ssuliman1@bwh.harvard.edu

Affiliations:

1. Department of Neurology, Massachusetts General Hospital, Harvard Medical School, Charlestown, Boston, MA
2. Department of Medicine, Harvard Medical School, Boston, MA
3. Mass General Brigham Innovation, Boston, MA
4. Cardiology Division, Massachusetts General Hospital, Charlestown, MA
5. Department of Neurosurgery, Brigham and Women's Hospital, Harvard Medical School, Boston, MA
6. Department of Medical Oncology and Center for Cancer-Genome Discovery, Dana-Farber Cancer Institute, Boston, MA
7. Department of Pathology, Harvard Medical School, Boston, MA

8. Center for Regenerative Medicine, Massachusetts General Hospital, Boston, MA
9. Harvard Stem Cell Institute, Cambridge, MA
10. Department of Psychiatry, Massachusetts General Hospital, Boston, MA
11. Wyss Institute for Biologically Inspired Engineering, Harvard University, Boston, MA
12. Department of Pathology, Brigham and Women's Hospital, Harvard Medical School, Boston, MA
13. Sackler School of Biomedical Sciences, Tufts University School of Medicine, Boston, MA
14. Division of Rheumatology, Inflammation and Immunity, Brigham and Women's Hospital, Boston, MA
15. Medical Diagnostic Technology Evaluation, LLC, Carlisle, MA
16. Center for Systems Biology, Massachusetts General Hospital, Boston, MA
17. Department of Pathology, Massachusetts General Hospital, Boston, MA
18. Wellman Center for Photomedicine, Massachusetts General Research Institute, Boston, MA
19. Department of Dermatology, Massachusetts General Hospital, Boston, MA
20. Evergrande Center for Immunologic Diseases, Brigham and Women's Hospital, Boston, MA
21. Cardiovascular Division, Department of Medicine, Brigham and Women's Hospital
22. Functional Genomics Laboratory, Channing Division of Network Medicine, Brigham and Women's Hospital, Boston, MA
23. Center for Cancer Research, Massachusetts General Hospital, Harvard Medical School, Charlestown, MA
24. Division of Nephrology and Endocrine Unit Department of Medicine, Massachusetts General Hospital, Boston, MA
25. Cancer Center Protocol Office, Massachusetts General Hospital, Boston, MA
26. Division of Preventative Medicine, Brigham and Women's Hospital, Harvard Medical School, Boston, MA
27. Radiology and pathology, Massachusetts General Hospital, Harvard Medical School, Boston, MA
28. Brigham Research Institute, Brigham and Women's Hospital, Boston, MA
29. Immunology Program, Harvard Medical School, Boston, MA
30. Cellular Immunotherapy Program, Cancer Center, Massachusetts General Hospital, Boston, Massachusetts, USA
31. Department of Radiation Oncology, Massachusetts General Hospital, Harvard Medical School, Boston, MA
32. Folia Health, Inc., Cambridge, MA

33. Vincent Center for Reproductive Biology, Department of Obstetrics and Gynecology, Massachusetts General Hospital, Boston, MA
34. Department of Biology, Northeastern University, Boston, MA
35. College of Science, Northeastern University, Boston, MA
36. Mass General Brigham COVID Center for Innovation, Diagnostics Accelerator, Boston, MA

Supplementary Methods:

Analysis

Computational analyses were performed in R/markdown. LFA evaluation reports are available (http://publicdata.omics.kitchen/Projects/MGBCCI/LFA/VendorReports/). Formulae for computing performance metrics and confidence intervals are provided below. IgG/IgM bands were called via visual inspection by two experienced human operators. Invalid LFAs (e.g. missing a control band) were excluded entirely from further analysis and the sample was not re-run.

A score was computed for each sample/assay/antibody combination, using the following algorithm:

- Each of N operators reading the LFA were assigned a weight of 1/N to final score
- If an operator observed a band, the score would increase by 1/N. If the operator saw no band, the score would decrease by 1/N

This process was performed separately for the IgG and IgM channels, with an overall score produced for each antibody. This per-sample/per-antibody score thus has the following values: -1 (all operators agree: no band), 0 (operators disagree), and +1 (all operators agree: band observed). Given that COVID-19 remains, even in late 2020, a low-prevalence disease, we apply a conservative case definition (score ≥0), where discordant operator readings (score=0) are classed as negative for presence of the antibody, in order to favor specificity. A combined IgG and IgM score was computed as the average of the two individual scores: (IgG+IgM)/2.

Reproducibility analysis used the same scoring system above. Tests of the COVID+ pool that received a score >0 were called "correct". COVID- tests were ones that received a score <0. A score of 1 or -1 was called "consistent", with all other scores indicating disagreement between operators called as "inconsistent." These two classifications were concatenated to produce the outcomes "correct consistent", where both operators agreed on the correct outcome, "incorrect consistent," where both operators agreed on the incorrect outcome, and "inconsistent," where operators disagreed on the outcome. The outcome of each individual test was plotted as a proportion of total tests of each pool on each of the two days of testing.

Webapp

We developed an interactive web-application (https://covid.omics.kitchen) to help visualize false positive and false negative LFA readings based on test accuracy and disease prevalence. We incorporate data from the 20 LFAs reported here as well as the 6 LFAs evaluated by Whitman, et al[16]. County, State, and US-wide disease rates (cumulative numbers of individuals confirmed to have been infected since the start of the outbreak) are pulled dynamically from the New York Times github repository (https://github.com/nytimes/covid-19-data). The webapp is implemented in d3.js JavaScript and hosted on Amazon Web Services AWS/Amplify.

Formula:

Sensitivity

- $n = TP + FN$
- $sens = \frac{TP}{n}$
- $SE_{sens} = \sqrt{\frac{sens(1-sens)}{n}}$
- $CI_{95\%} = (sens - z_{\frac{\alpha}{2}} * SE_{sens} - \frac{1}{2n}, sens + z_{\frac{\alpha}{2}} * SE_{sens} + \frac{1}{2n})$

Specificity

- $n = TN + FP$
- $spec = \frac{TN}{n}$
- $SE_{spec} = \sqrt{\frac{spec(1-spec)}{n}}$
- $CI_{95\%} = (spec - z_{\frac{\alpha}{2}} * SE_{spec} - \frac{1}{2n}, spec + z_{\frac{\alpha}{2}} * SE_{spec} + \frac{1}{2n})$

PPV

- $n = TP + FP$
- $PPV = \frac{TP}{n}$
- $SE_{PPV} = \sqrt{\frac{PPV(1-PPV)}{n}}$
- $CI_{95\%} = (PPV - z_{\frac{\alpha}{2}} * SE_{PPV} - \frac{1}{2n}, PPV + z_{\frac{\alpha}{2}} * SE_{PPV} + \frac{1}{2n})$

NPV

- $n = TN + FN$
- $NPV = \frac{TN}{n}$
- $SE_{NPV} = \sqrt{\frac{NPV(1-NPV)}{n}}$
- $CI_{95\%} = (NPV - z_{\frac{\alpha}{2}} * SE_{NPV} - \frac{1}{2n}, NPV + z_{\frac{\alpha}{2}} * SE_{NPV} + \frac{1}{2n})$

Supplementary Table 1: LFA commercial kit information, sample requirements, and protocol details

Manufacturer	Lot Number	Biological Target	Sample Requirement	IgG/IgM Cassette Structure	Total input volume (uL)	Incubation time (minutes)
API_V1	CoV1252004C	N and S	whole blood, plasma, serum	Same strip	10	15-20
API_V2	COV1252008B	N and S	whole blood, plasma, serum	same strip	10	15-20
BioHit	SA200401	N and S	venous whole blood, plasma, serum	Same strip	10	15-20
BTNX	I2004027	N and S	whole blood, plasma, serum	Same strip	5	15
Camtech	CAM240420	S	plasma, serum	same strip	10	15
CareHealth	G070720	N	venous whole blood, plasma, serum	Same strip	5	15-Oct
Cellex	20200503WI5515C025	N and S	venous whole blood, plasma, serum	Same strip	10	15-20
Edinburgh	2000798A	N and S	whole blood (capillary and venous)	Same strip	20	10
Genobio	VMG200331	N and S	venous whole blood, plasma, serum	Same cassette, separate strips	20	10
InTec	ITP6002-TC25/GJ20030288	N and S	whole blood (venous and fingerstick), plasma, serum	Same cassette, separate strips	20	15-20
KHB	423200332	N	fresh whole blood, plasma, serum	Same strip	10	15
Livzon	CK2004240410	N and S	venous whole blood, plasma, serum	Separate cassettes	20	15-Jan
Lumiquick	2004219	N and S	venous whole blood, plasma, serum	Same strip	2	15
Oranoxis	RC-0220	S	plasma, serum	Same strip	10	15
Ozo	P2002	S	whole blood (capillary and venous), plasma, serum	Same cassette, separate strips	20	10
Pharmatech	D00647	N	whole blood (venous and fingerstick), plasma, serum	Same strip	10	10
RayBiotech_V1	501202955	N	whole blood (venous and fingerstick), serum	Same cassette, separate strips	25	10
RayBiotech_V2	715202954	N	whole blood (venous and fingerstick), serum	Same cassette, separate strips	25	10
U2U	172004-01	N and S	whole blood (venous and fingerstick), plasma, serum	Same strip	10	15
VivaCheck	SU2005001	N and S	whole blood (venous and fingerstick), serum, heparin plasma	Same strip	10	15

Supplementary Table 2: IFU clarity rubric. Kits were assigned one point for each criteria listed if met

	**Criteria for IFU clarity**
1	Includes explanation of intended use
2	Storage conditions of provided kit components are listed
3	Sample requirements and collection methods (e.g. whole blood, serum, plasma, equilibration temperature, etc.)
4	Storage information for specimens
5	Precautions/provides warnings and conditions to avoid (e.g. do not use expired cassettes, etc.)
6	Test procedure: temperature specified for running sample and cassette
7	Test procedure: clearly states when cassette should be read
8	Test procedure: clearly states valid time window (e.g. test results invalid after a certain interval of time)
9	Test procedure: provides visual diagrams for protocol steps
10	Provides instruction on how to proceed if results are invalid (e.g. repeat test, or increase incubation time)
11	Includes a rubric for interpretation of results
12	Provides visual diagrams or cassette images to assist with interpretation of results (has to include at least an example of a positive and negative readout)
13	Clearly describes correct volume to pipette (e.g. marked pipette, markings explained in the IFU, or dropwise measurement)
14	Complete instructions provided for lancets or other accessories included for use (e.g. instructions present for all kit components)

Supplementary Table 3. Usability ratings and description of kit components.

Manufacturer	Pipette type	If pipette provided, material	If pipette provided: usability (e.g. volume markings?)	Buffer type (dropper, aliquot)	Rater 1	Rater 2	Rater 3	IFU Clarity Final Ratings	Additional materials provided	Notes
API_V1	plastic dropper	Plastic	no markings	dropper	11	11	12	12		
API_V2	None included	NA	NA	dropper	12	13	12	12	lancets, alcohol pads, bandaids	Cassette is double packaged in 2 sealed pouches, no pipette is provided
BioHit	Plastic dropper	Plastic	no markings	dropper	13	14	14	14		
BTNX	Plastic dropper	Plastic	volume markings	dropper	12	13	13	12		Outcome call requires interpretation of color - difficult for color blind?
Camtech	Plastic dropper	Plastic	no markings	dropper	9	12	10	12		Provides useful information on how long it takes materials to reach room temp.
CareHealth	Plastic dropper	Plastic	no markings	dropper	13	14	13	13		Instructions say add 10uL of whole blood, but doesn't show what that looks like in the provided pipette
Cellex	Plastic dropper	Plastic	no markings	dropper	11	13	13	13		Provides visual ref to show that even faint bands can be positive.<br>Requires 10ul whole blood but doesn't show what that looks like in the plastic pipette
Edinburgh	Plastic dropper	Plastic	volume markings	aliquot	10	13	10	10	cotton swab, lancet, band-aid, alcohol pad	Requires 10ul whole blood doesn't show how much that is in plastic pipette
Genobio	Plastic dropper	Plastic	no markings	dropper	8	11	9	10		Not clear if requires<br>10ul sample in total,<br>2 drops in total,<br>or if the cassette should be "standing"
InTec	Plastic dropper	Plastic	no markings	dropper	14	14	14	14	lancet, alcohol pads	
KHB	Plastic dropper	Plastic	no markings	dropper	13	12	12	11	lancet, alcohol pads	The procedure contains typos, including:<br>"add 10ul of plasma **and perum** specimen"
Livzon	Capillary pipette	Glass	volume markings	dropper	13	12	13	13		Large range of time in which to interpret samples (1-15 mins)
Lumiquick	Capillary pipette	Plastic	volume markings	dropper	14	14	14	14		
Oranoxis	Capillary pipette	Plastic	no markings	dropper	8	9	8	7		Conflicting guidance on time to read, no specific mentions of sample requirements/ storage
Ozo	Plastic dropper	Plastic	no markings	dropper	11	12	12	12	cotton swab, lancet, alcohol pads	No visual reference<br>for invalid cassette
Pharmatech	Plastic dropper	Plastic	no markings	dropper	14	14	14	14	lancets, alcohol pads	
RayBiotech_V1					13	13	13			
RayBiotech_V2	Plastic dropper	Plastic	volume markings	aliquot	14	13	13	13		
U2U	Capillary pipette	Plastic	volume markings	dropper	14	14	14	14		
VivaCheck	Plastic dropper	Plastic	no markings	dropper	11	12	11	10		No separate sectionfor sample requirements or collection

Supplementary Table 4. Clinical information for the COVID-positive individuals whose plasma was used in this study. Individuals are broken out by the number of days between symptom onset and blood collection

Interval between symptom onset and blood collection	7-14 days	15-21 days	22-28 days	29+ days	Overall
	n = 9	n = 18	n = 16	n = 13	n=56
**Sex**					
Female	6 (66.7%)	10 (55.6%)	11 (68.8%)	4 (30.8%)	31 (55.4%)
Male	3 (33.3%)	8 (44.4%)	5 (31.2%)	9 (69.2%)	25 (44.6%)
**Age**					
Mean (Standard Deviation)	72.0 (24.0)	53.0 (15.8)	52.9 (19.9)	64.7 (20.2)	58.7 (20.4)
Median [Min, Max]	85	49	54.5	67	57.5
	[30, 98]	[30, 84]	[24, 88]	[29, 94]	[24, 98]
**Race**					
Asian	1 (11.1%)	1 (5.6%)	1 (6.2%)	0 (0%)	3 (5.4%)
Black	5 (55.6%)	5 (27.8%)	2 (12.5%)	4 (30.8%)	16 (28.6%)
LatinX	1 (11.1%)	3 (16.7%)	1 (6.2%)	0 (0%)	5 (8.9%)
Unknown/Other	1 (11.1%)	1 (5.6%)	2 (12.5%)	4 (30.8%)	8 (14.3%)
White	1 (11.1%)	8 (44.4%)	10 (62.5%)	5 (38.5%)	24 (42.9%)
**Primary COVID PCR test**					
Cepheid Xpert Xpress SARS-CoV-2	1 (11.1%)	0 (0%)	1 (6.2%)	0 (0%)	2 (3.6%)
Panther Fusion SARS-CoV-2	8 (88.9%)	17 (94.4%)	12 (75.0%)	12 (92.3%)	49 (87.5%)
In house LDT at the Broad Institute	0 (0%)	1 (5.6%)	3 (18.8 %)	1 (7.7%)	5 (8.9%)
**Severity**					
Deceased	0 (0%)	0 (0%)	4 (25%)	0 (0%)	4 (7.1%)
Hospitalized, not ICU	7 (77.8%)	13 (72.2%)	2 (12.5%)	2 (15.4%)	24 (42.9%)
ICU	0 (0%)	0 (0%)	2 (12.5%)	2 (15.4%)	4 (7.1%)
ICU (intubated)	0 (0%)	1 (5.6%)	1 (6.2%)	1 (7.7%)	3 (5.4%)
ICU (intubated) - Recovered	0 (0%)	3 (16.7%)	1 (6.2%)	7 (53.8%)	11 (19.6%)
ICU (no intubation) - Recovered	1 (11.1%)	0 (0%)	2 (12.5%)	0 (0%)	3 (5.4%)
Outpatient	1 (11.1%)	1 (5.6%)	4 (25%)	1 (7.7%)	7 (12.5%)

Supplementary Table 5. LFA Limits of detection estimated from anti-spike antibodies concentrations measured by Simoa; amounts reflect lowest antibody concentrations in plasma samples that show a positive band

LFA manufacturer	LoD IgG (μg/mL)	LoD IgM (μg/mL)
API	4.4	5111.7
API (v2)	4.4	6.1
BioHit	4.4	0.6
BTNX	8.7	7.5
Camtech	27.5	83.8
CareHealth	1.5	7.5
Cellex	1.5	5111.7
Edinburgh	1453.8	5111.7
Genobio	14318.7	5111.7
InTec	27.5	2940.5
KHB	1.5	2940.5
Lumiquick	0.1	5111.7
Oranoxis	14318.7	10.9
OZO	14318.7	5111.7
Phamatech	27.5	42.4
Ray Biotech	14318.7	638.4
Ray Biotech (v2)	6.5	29.8
U2U	14318.7	5111.7
Vivachek	1.5	3.4
Zhuhai Livzon	27.5	29.8
